# Microencapsulated oregano essential oil improves growth performance, carcass characteristics, and meat quality in broiler chickens

**DOI:** 10.14202/vetworld.2026.3457-3473

**Published:** 2026-08-11

**Authors:** Liang Yan, Cao Leping, Tao Lei, Liang Jianping, Xia Zhi, Jia Erling

**Affiliations:** 1 College of Agronomy, Henan Agricultural University, Zhengzhou 450046, China.; 2 Henan Shennong Genuine Medicinal Materials Co., Ltd., Zhengzhou 450040, China.

**Keywords:** antibiotic alternatives, broiler chickens, carcass characteristics, growth performance, meat quality, microencapsulation, oregano essential oil, phytogenic feed additives

## Abstract

**Background and Aim:**

The increasing restrictions on antibiotic growth promoters have intensified the search for safe and effective phytogenic alternatives for poultry production. Oregano (*Origanum vulgare L.*) essential oil (OEO) possesses antibacterial, antioxidant, anti-inflammatory, and immunomodulatory activities, but its high volatility and poor thermal stability limit practical applications. This study aimed to develop microencapsulated OEO and evaluate its effects on growth performance, carcass characteristics, and meat quality in broiler chickens.

**Materials and Methods:**

OEO microcapsules were prepared by complex coacervation using gelatin and gum Arabic as wall materials. Physicochemical characteristics were evaluated through encapsulation efficiency, oil loading capacity, particle size analysis, Fourier-transform infrared spectroscopy, scanning electron microscopy, thermogravimetric analysis, differential scanning calorimetry, and release behavior studies. A feeding trial involving 3,600 one-day-old Arbor Acres broilers was conducted for 42 days. Birds were randomly allocated to four dietary treatments containing 0, 80, 120, or 160 mg/kg microencapsulated OEO. Growth performance, slaughter traits, meat quality parameters, and proximate composition of breast and thigh muscles were determined.

**Results:**

The optimized microcapsules exhibited an encapsulation efficiency of 84.2%, a product yield of 82.37%, and a mean particle size of 151 μm. Structural and thermal analyses demonstrated effective encapsulation and enhanced thermal stability with sustained-release properties. Dietary supplementation with microencapsulated OEO improved broiler performance in a dose-dependent manner. Birds receiving 160 mg/kg supplementation exhibited significantly greater final body weight and average daily gain, together with reduced feed-to-gain ratio and mortality rate (p < 0.05). Breast muscle yield increased, whereas abdominal fat percentage decreased significantly (p < 0.05). Supplementation also improved meat quality by increasing muscle pH, reducing drip loss, enhancing redness, and decreasing lightness. Furthermore, breast muscle crude protein content increased, whereas crude fat content in breast and thigh muscles decreased significantly (p < 0.05).

**Conclusion:**

Microencapsulation using gelatin and gum Arabic effectively enhanced the stability and controlled-release properties of OEO. Dietary inclusion of 160 mg/kg microencapsulated OEO improved growth performance, carcass characteristics, and meat quality while reducing fat deposition in broilers. These findings indicate that microencapsulated OEO represents a promising phytogenic feed additive and a potential alternative to antibiotic growth promoters in sustainable poultry production.

## INTRODUCTION

In intensive livestock production, the long-term misuse of antibiotics as growth promoters and prophylactic additives has raised several concerns, including the emergence of bacterial resistance, excessive drug residues, disruption of the gut microbiota, and environmental contamination. These issues pose substantial threats to animal health, food safety, and public health. A positive relationship exists between antibiotic consumption and the prevalence of bacterial resistance, and the continued overuse of antibiotics has hindered the sustainable development of the poultry industry. Antimicrobial resistance has become a major global challenge, and the development of new antibiotics alone is insufficient to counteract its escalating impact. In 2006, the European Union implemented a complete ban on the use of antibiotics as growth-promoting feed additives. In 2017, the United States introduced the Veterinary Feed Directive, which requires that medically important antibiotics used in food-producing animals be prescribed by veterinarians and restricted to therapeutic, control, or disease-prevention purposes. To mitigate antimicrobial resistance originating from livestock, the Chinese Ministry of Agriculture and Rural Affairs issued Announcement No. 194, mandating the complete withdrawal of growth-promoting drug feed additives beginning in 2020. Furthermore, the National Action Plan for Reducing the Use of Veterinary Antimicrobials continues to promote the “ban, restriction, and reduction” strategy for antimicrobial use in animal production, encouraging the development and application of safe and environmentally friendly alternatives. In 2020, China terminated the production, importation, and use of all growth-promoting drug feed additives, except traditional Chinese medicines, thereby entering the era of antibiotic-free feed production. Consequently, the development of plant-derived antibiotic alternatives has become an important research focus. Among these alternatives, plant essential oils have attracted considerable attention because of their safety and potential applications in livestock and poultry production [1].

Oregano (*Origanum vulgare L.*), a member of the family *Lamiaceae*, is native to Europe, northern Africa, and temperate regions of Asia, and is widely distributed throughout the Mediterranean region, particularly in Turkey. Traditionally, oregano has been used as both a spice and a medicinal herb to manage influenza, fever, vomiting, acute gastroenteritis, and indigestion. Oregano essential oil (OEO), which is extracted from the aerial parts of *O. vulgare* L., contains several bioactive constituents, among which carvacrol and thymol are the major components. These compounds possess antibacterial, antioxidant, anti-inflammatory, and immunomodulatory activities. In recent years, natural essential oils have emerged as promising alternatives to conventional antibiotics and have become a major area of research in the feed and veterinary industries [2–4]. The Chinese Ministry of Agriculture and Rural Affairs has included thymol and carvacrol, the principal active components of OEO, in the Catalog of Feed Additive Varieties [5]. Owing to their natural origin, high biological efficacy, low residue levels, and reduced risk of antimicrobial resistance, OEO-based products are considered attractive alternatives to antibiotics. They can improve poultry health and productivity through antibacterial and anti-inflammatory effects, protection of intestinal mucosa, enhancement of immune responses, and improved feed utilization efficiency. These properties align with national strategies to promote green farming practices, antibiotic-free production, and food safety, making OEO a promising feed additive for sustainable poultry production.

Despite these advantages, the practical application of OEO remains limited because of its physicochemical instability. The active compounds are highly volatile and thermolabile, resulting in rapid degradation and loss of antimicrobial activity during storage and feed processing [6]. High-temperature processing conditions further accelerate the volatilization and deterioration of active ingredients, thereby reducing their biological effectiveness and limiting their commercial use [7]. Microencapsulation technology has emerged as an effective strategy to improve the stability, controlled-release, and bioavailability of essential oils, thereby overcoming some of the limitations associated with conventional formulations [8].

Previous studies have demonstrated the biological activities of OEO and its beneficial effects on poultry health; however, most investigations have focused either on the chemical characterization of the essential oil or on its direct dietary supplementation. Information on the development of stable microencapsulated OEO formulations and their simultaneous evaluation of physicochemical properties, sustained-release behavior, growth performance, carcass characteristics, and meat quality in broiler chickens remains limited. Moreover, comprehensive studies integrating encapsulation technology with practical animal production outcomes are scarce. Therefore, there is a need to establish an effective microencapsulation system to enhance the stability and functionality of OEO and to validate its biological efficacy under commercial broiler production conditions [9, 10].

Accordingly, this study aimed to prepare OEO microcapsules using complex coacervation technology and to characterize their physicochemical properties, thermal stability, and sustained-release behavior. In addition, the study evaluated the effects of dietary supplementation with microencapsulated OEO on growth performance, carcass characteristics, meat quality, and muscle nutritional composition in broiler chickens. By integrating encapsulation technology with animal production performance, this study sought to provide scientific evidence supporting the application of microencapsulated OEO as a safe and effective phytogenic feed additive and a potential alternative to antibiotic growth promoters in sustainable poultry production.

## MATERIALS AND METHODS

### Ethical approval

All experimental procedures involving animals were reviewed and approved by the Animal Care and Use Committee of Henan Agricultural University, Zhengzhou, China (Approval No. HNND2020031012). The approved protocol encompassed the experimental procedures and welfare considerations implemented throughout the study. The experiment was conducted in accordance with institutional guidelines for the ethical use of animals in research and complied with internationally accepted principles for animal welfare and the 3Rs concept (Replacement, Reduction, and Refinement). Continuous veterinary oversight was maintained during the experimental period, and all procedures were performed by trained personnel to ensure humane treatment of the birds and to minimize pain, distress, and unnecessary suffering. Before commencement of the study, informed consent was obtained from the farm owners for the use of animals and collection of research data.

### Study period and location

The experiment was conducted from March 2025 to February 2026 at the Weilai Agricultural Breeding Farm in Ye County (33°08′N to 34°20′N, 112°14′E to 113°45′E), Henan Province, China. Preparation and characterization of OEO microcapsules, as well as subsequent feeding trials, were conducted under controlled laboratory and farm conditions. The feeding trial lasted for 42 days and consisted of a starter phase (1–21 days) and a finisher phase (22–42 days).

### Study design

A completely randomized design was employed in this study. A total of 3,600 healthy 1-day-old Arbor Acres (AA) broilers with similar initial body weights were randomly allocated to four treatment groups, each with six replicates of 150 birds. The control group (CK) received a basal diet without feed additives, whereas groups A1, A2, and A3 received dietary supplementation with microencapsulated OEO at concentrations of 80, 120, and 160 mg/kg, respectively. The supplementation levels were selected based on previous reports, the sustained-release properties of the microcapsules, and results obtained from preliminary experiments. The dose-response design was intended to determine the optimal inclusion level and evaluate the biological effects of microencapsulated OEO in broilers.

### Determination of the maximum absorption wavelength of OEO

0.3 μL of OEO was accurately weighed, dissolved in anhydrous ethanol, and diluted to obtain a solution of the desired concentration. Using anhydrous ethanol as the blank control, full-wavelength scanning was performed with a UV–visible spectrophotometer over the wavelength range of 200–1100 nm to determine the maximum absorption wavelength of OEO.

### Preparation of OEO microcapsules

OEO microcapsules were prepared by the complex coacervation method. Gelatin and gum Arabic solutions were prepared separately and then uniformly mixed. OEO was then added to the mixed solution at a core-to-wall ratio of 3:1. The emulsifier WJE-880 was incorporated at a concentration of 0.1%.

The resulting mixture was homogenized by centrifugation at 4,025 × *g* for 3 min, after which the pH was adjusted to 4.0 using 10% (v/v) glacial acetic acid. The mixture was stirred at 7 × *g* for 30 min and then cooled to approximately 10°C in an ice-water bath. Subsequently, the pH was adjusted to 6.0 using 2.5 mol/L sodium hydroxide solution, and the reaction temperature was maintained at 50°C.

Transglutaminase 5% was added to the system, and stirring was continued for 3 h to complete cross-linking and curing. The resulting suspension was allowed to stand and then vacuum-filtered to obtain wet microcapsules. The wet microcapsules were evenly spread in Petri dishes, pre-cooled at 4°C for 24 h, and freeze-dried for 36 h to obtain solid OEO microcapsules.

### Encapsulation efficiency and product yield

Anhydrous ethanol was used as the solvent to prepare OEO standard solutions with concentrations ranging from 0.5 to 2.5 μL/mL. The absorbance values were measured at 277 nm, and the following standard calibration equation was obtained:


\[
Y=1.342X+0.0103 \left({R}^{2}=0.9998\right)
\]

To determine the amount of encapsulated OEO, 0.1 g of microcapsules was placed into a 30-mL centrifuge tube containing 10 mL of anhydrous ethanol. The mixture was sonicated for 20 min and allowed to stand for 12 h, followed by centrifugation at 699 × *g* for 15 min. Subsequently, 1 mL of the supernatant was transferred into a 25-mL volumetric flask and diluted to volume with anhydrous ethanol. The absorbance at 277 nm was measured, and the OEO content in the microcapsules was calculated from the calibration curve.

The encapsulation efficiency and product yield were calculated according to the following equations [11]:


\[
\begin{matrix} & Encapsulation efficiency = \frac{A}{M} \times 100 & & \end{matrix}
\]

\[
\begin{matrix} & Yield = \frac{m}{B+M } \times 100 & & \end{matrix}
\]

where *A* represents the amount of OEO encapsulated in the microcapsules (g), *M* is the initial amount of OEO added (g), *m* is the mass of the obtained microcapsules (g), and *B* is the amount of wall materials added (g).

### Determination of physicochemical properties of OEO microcapsules

**Determination of encapsulation efficiency and oil loading capacity:** The encapsulation efficiency of the microcapsules was defined as the ratio of the mass of core material retained in the microcapsules to the amount of core material initially present in the emulsion. Oil loading capacity was defined as the mass ratio of core material to wall material in the microcapsules.

Encapsulation efficiency and oil loading capacity were calculated according to the following equations [12]:


\[
\begin{matrix} & Encapsulation efficiency = \frac{({V}_{1} - {V}_{2})\rho }{{V}_{1}} \times 100 & & \end{matrix}
\]

\[
\begin{matrix} & Oil loading capacity = \frac{({V}_{1} - {V}_{2})\rho }{ m-{V}_{1}\rho } \times 100 & & \end{matrix}
\]

where *V1* is the total oil volume of the microcapsules (mL), *V2* is the surface oil volume (mL), *m* is the mass of the microcapsule powder (g), and ρ is the density of OEO (g/mL).

**Determination of total oil content:** A 0.1-g sample of freeze-dried OEO microcapsule powder was weighed and immersed in 10 mL of anhydrous ethanol. The mixture was subjected to ultrasonic treatment using an ultrasonic cell disruptor at 200 W for 10 min at 20 °C, then allowed to stand for 2 h. After filtration, the filtrate was adjusted to a final volume of 20 mL. The total oil content of the microcapsules was subsequently determined by high-performance liquid chromatography.

**Particle size measurement:** The particle size distribution of the microcapsules was determined using a laser particle size analyzer with distilled water as the dispersing medium. Instrument parameters were set as follows: refractive index of the dispersant, 1.33; refractive index of the sample, 1.59; sample absorbance, 0%; and analysis mode, general model.

**Fourier-transform infrared spectroscopy (FTIR) analysis:** FTIR was performed to characterize the chemical structure of the samples. Appropriate amounts of sample and potassium bromide (KBr) powder were thoroughly mixed and finely ground. The mixture was compressed into transparent pellets using a tableting machine and scanned in the range of 500–4000 cm⁻¹ with a spectral resolution of 0.09 cm⁻¹ [13].

### Scanning electron microscopy (SEM) observation

For morphological analysis, conductive adhesive tape was attached to the specimen holder, and freeze-dried microcapsule powder was uniformly distributed on the tape surface. Excess powder was removed by gentle air blowing. After sputter coating with gold, the surface morphology of the microcapsules was examined and photographed using a scanning electron microscope.

### Thermogravimetric analysis (TGA)

The thermal decomposition characteristics of the samples were evaluated using a thermogravimetric analyzer. The analysis was performed over the temperature range of 25–500 °C at a heating rate of 20 °C/min in a nitrogen atmosphere at a flow rate of 30 mL/min. The resulting thermogravimetric curves were used to characterize the thermal stability and decomposition behavior of the samples.

### Differential scanning calorimetry (DSC)

DSC was employed to determine the glass transition temperature (*Tg*) of the samples. Samples were sealed in DSC pans, and an empty pan was used as the reference. Measurements were conducted over the temperature range of 0–100 °C at a heating rate of 10 °C/min [14].

### Release behavior of OEO microcapsules

Equal amounts of OEO microcapsules were stored under different environmental conditions to investigate their release behavior. The storage conditions included exposure to natural light or darkness (25 °C, aerobic conditions); storage at 4 °C or 25 °C under dark, aerobic conditions; and storage under aerobic or anaerobic conditions at 25 °C in the dark. The OEO release rate was determined every 5 days, and all measurements were conducted in triplicate.

The release rate was calculated according to the following equation:


\[
\begin{matrix} & Release rate = \frac{{ V}_{1}-{V}_{t} }{{V}_{1}} \times 100 & & \end{matrix}
\]

where *V1* represents the initial volume of OEO in the microcapsules (mL), and *Vt* represents the volume of OEO remaining in the microcapsules at day *t* (mL).

### Growth performance and meat quality of broilers [15]

A total of 3,600 healthy one-day-old AA broilers with similar initial body weights were randomly allocated to four treatment groups. Each treatment consisted of six replicates, with 150 birds per replicate. The control group (CK) received the basal diet without additives, whereas birds in groups A1, A2, and A3 received diets supplemented with OEO microcapsules at levels of 80, 120, and 160 mg/kg, respectively.

The supplementation levels were selected based on the effective dosage ranges reported in previous domestic and international studies, the sustained-release characteristics of microencapsulation, and the results of preliminary experiments. Preliminary studies demonstrated that 80–160 mg/kg represented the critical dosage range for exerting beneficial effects on broilers. Supplementation below this range had limited effects, whereas higher levels did not yield further improvement. Therefore, graded supplementation levels were adopted to elucidate the dose-response relationship and identify the optimal inclusion level.

The experiment was conducted at the Weilai Agricultural Breeding Farm in Ye County, Henan Province, China, and lasted 42 days. The experimental period was divided into a starter phase (1–21 d) and a finisher phase (22–42 d).

During the starter phase, birds in the control group received the basal diet, whereas birds in the treatment groups received diets supplemented with OEO microcapsules, with the supplementation level increased by 25 mg/kg daily from day 7 until the target dosage for each treatment was achieved. During the finisher phase, the control group (CK) continued to receive the basal diet, whereas groups A1, A2, and A3 received diets containing OEO microcapsules at 80, 120, and 160 mg/kg, respectively. Fresh diets were prepared daily, and the microcapsules were mixed immediately before feeding.

Except for dietary treatments, all management practices were identical across groups and followed the farm’s routine procedures. Birds were maintained at 30–35 °C during the first week, after which the temperature was gradually reduced by approximately 2 °C per week until reaching 25 ± 1 °C. Relative humidity was maintained at 45%–55%. Routine vaccination and husbandry procedures were performed in accordance with the farm management program. Lighting was interrupted for 1 h daily, and birds had *ad libitum* access to feed and water throughout the experiment.

### Experimental diets

The basal diet consisted of a self-formulated mash feed, prepared with minor modifications, in accordance with the Feeding Standard for Chickens of China (NY/T 33–2004). For the 1–21-day starter diet, metabolizable energy (ME) was increased by 0.42 MJ/kg, crude protein (CP) by 0.55%, calcium reduced by 0.05%, total phosphorus increased by 0.03%, available phosphorus increased by 0.03%, lysine increased by 0.12%, and methionine increased by 0.06%. For the 22–42-day finisher diet, ME was increased by 0.41 MJ/kg, CP by 0.05%, calcium reduced by 0.02%, total phosphorus increased by 0.01%, available phosphorus increased by 0.02%, lysine increased by 0.10%, and methionine increased by 0.03%. Separate diets were formulated for the starter phase (1–21 d) and the finisher phase (22–42 d). The ingredient composition and nutrient levels of the basal diets are presented in Table 1.

### Determination of growth performance

Feed intake was recorded throughout the experiment. Body weights of broilers in each replicate were measured on days 21 and 42. Bird health status and the numbers of dead and culled birds were monitored and recorded daily.

The following performance indices were calculated:


\[
Average daily feed intake (ADFI) = \frac{Total feed consumption}{ Trial days \times Number of broilers }
\]

\[
Average daily gain (ADG) = \frac{Final body weight - Initial body weight}{Trial days}
\]

\[
Feed-to-gain \frac{F}{G} =\frac{ADFI}{ADG}
\]

\[
Mortality and culling rate (\%) = \frac{Number of dead and culled birds}{Total number of birds} \times 100
\]

**Table 1:** Composition and nutritional levels of basal diets

**Item**	**1–21 days of age**	**22–42 days of age**
Diet composition (%)		
Corn	55.77	57.15
Soybean meal	26.37	25.62
Wheat middlings	5.00	5.00
Corn gluten meal	3.00	3.00
Fish meal	3.00	0.00
Calcium hydrogen phosphate	2.00	1.84
Soybean oil	2.00	4.50
Limestone powder	1.00	0.96
Sodium chloride	0.30	0.30
Premix	1.00	1.00
Methionine	0.11	0.10
Lysine (95%)	0.35	0.43
Choline chloride	0.10	0.10
Nutritional levels		
Metabolizable energy (MJ/kg)	12.55	12.96
Crude protein (%)	21.55	19.05
Calcium (%)	0.95	0.88
Total phosphorus (%)	0.68	0.61
Available phosphorus (%)	0.48	0.42
Lysine (%)	1.12	1.00
Methionine (%)	0.51	0.45

Each kilogram of premix provided 15,000 IU vitamin A, 3,000 IU vitamin D, 200 IU vitamin E, 3.5 mg vitamin B6, 2.0 mg vitamin K3, 2.0 mg vitamin B1, 0.01 mg vitamin B12, 200 mg iron, 80 mg manganese, 60 mg zinc, 8 mg copper, 0.45 mg iodine, 0.35 mg selenium, 35 mg folic acid, 10 mg calcium pantothenate, 6 mg riboflavin (vitamin B2), and 0.30 mg biotin. The crude protein, calcium, and total phosphorus values were determined experimentally, whereas the remaining nutritional values were calculated.

### Determination of slaughter performance

At the end of the experiment, 15 broilers with body weights close to the average value were randomly selected from each replicate for slaughter and carcass evaluation. Slaughter performance was determined according to the industrial standard *Terminology and Measurement Methods for Poultry Production Performance* (NY/T 823–2020).

The following parameters were calculated:


\[
Dressing percentage = \frac{Dressed weight}{Live weight before slaughter} \times 100
\]

\[
Semi-eviscerated percentage = \frac{Semi-eviscerated weight}{Live weight before slaughter} \times 100
\]

\[
Full-eviscerated percentage = \frac{Full-eviscerated weight}{Live weight before slaughter} \times 100
\]

\[
Breast muscle percentage = \frac{Breast muscle weight}{Full-eviscerated weight} \times 100
\]

\[
Thigh muscle percentage = \frac{Thigh muscle weight}{Full-eviscerated weight} \times 100
\]

### Determination of meat quality

Meat quality parameters, including pH, shear force, drip loss, and color characteristics, were determined in accordance with the industrial standard *Determination of Livestock and Poultry Meat Quality* (NY/T 1333–2007).

Following slaughter, carcasses were stored at 4 °C. The pH values of breast and thigh muscles were measured at 1 h (*pH₁ₕ*) and 24 h (*pH₂₄ₕ*) postmortem using a pH meter. Three measurements were obtained for each sample, and the mean value was used for analysis.

Meat color parameters, including lightness (L*), redness (a*), and yellowness (b*), were measured using a colorimeter at 1 h postmortem. Areas exhibiting congestion, fascia, or lesions were avoided during measurements. Three readings were obtained from each sample, and the average value was recorded.

Shear force was measured using a texture analyzer, with three replicates performed for each sample. Drip loss was determined by the hanging method. Briefly, meat samples were weighed, suspended in drip tubes, and stored at 4 °C for 24 h. After storage, the samples were removed, surface moisture was gently blotted off, and the samples were reweighed. Drip loss was subsequently calculated.

### Determination of conventional nutritional components in meat

Breast and thigh muscle samples were collected after slaughter for the analysis of chemical composition. Crude protein, crude fat, moisture, and ash contents were determined according to the National Food Safety Standards of China, including GB 5009.5–2016 for protein determination, GB 5009.6–2016 for fat determination, GB 5009.3–2016 for moisture determination, and GB 5009.4–2016 for ash determination.

### Statistical analysis

Experimental data were organized using Excel 2007 (Microsoft, Washington, USA). Statistical analyses were performed using DPS software (http://www.dpsw.cn). One-way analysis of variance was employed to evaluate treatment effects, and multiple comparisons among means were conducted using the least significant difference test. Data are expressed as mean ± standard deviation. Differences were considered statistically significant at *p* < 0.05, whereas* p* > 0.05 indicated the absence of statistically significant differences.

## RESULTS

### Maximum absorption wavelength of OEO

Full-wavelength scanning of OEO showed absorption peaks at 232 nm and 276 nm. However, the absorption band near 230 nm was attributed to anhydrous ethanol, which was used as the solvent. Therefore, the maximum absorption wavelength of OEO was determined to be 276 nm.

### Physicochemical properties and sustained-release performance of OEO microcapsules

The main active components detected in OEO microcapsules were thymol (23.88%) and carvacrol (3.64%). Under the optimized preparation conditions, the microcapsule yield reached 82.37%, while the encapsulation efficiency and oil loading capacity were 85.33% and 75.52%, respectively.

### Particle size of OEO microcapsules

**Figure 1:**
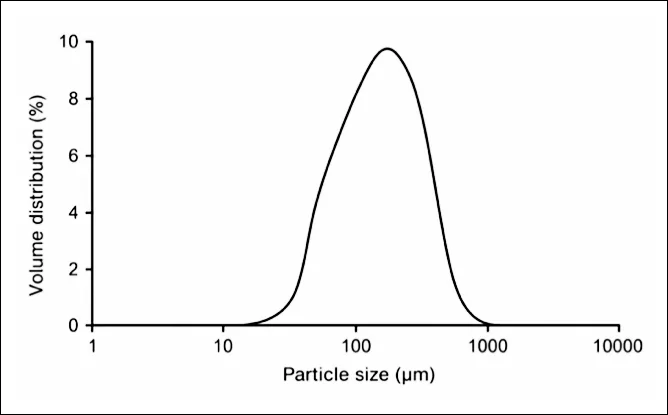
Particle size distribution of oregano essential oil microcapsules.

As shown in Figure 1, OEO microcapsules exhibited a uniform particle size distribution, with an average particle size of 151 μm.

### FTIR analysis

**Figure 2:**
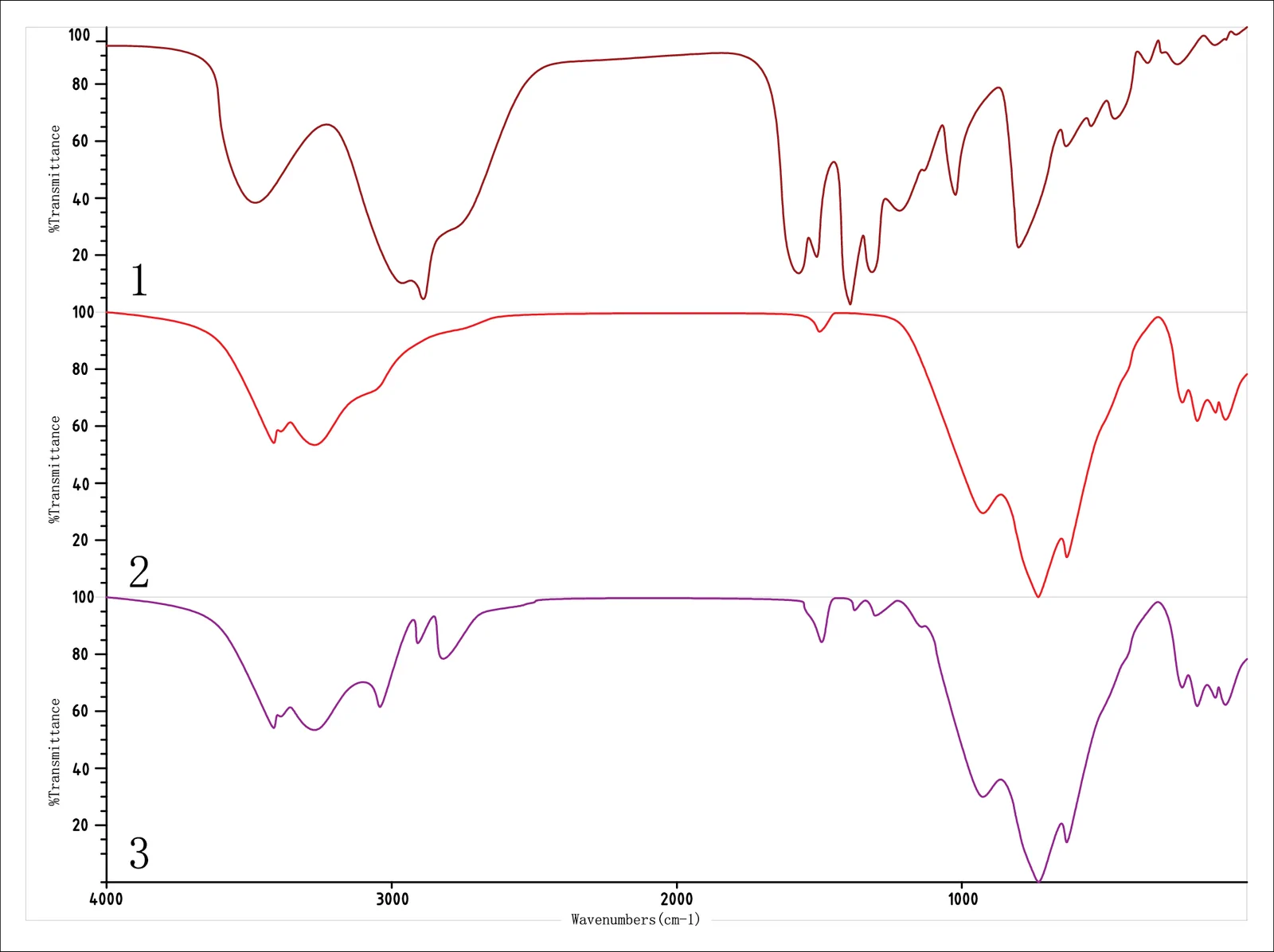
Fourier transform infrared spectroscopy of oregano essential oil (OEO) microcapsules. Curve 1: OEO; Curve 2: gum Arabic; Curve 3: OEO microcapsules.

As shown in Figure 2, the characteristic absorption peaks of OEO included the stretching vibration peak of the -OCH₂ group of flavonoids at 2900 cm⁻¹ and anhydride vibration peaks at 1620 cm⁻¹ and 1380 cm⁻¹. The attenuation of these anhydride peaks indicated that the microcapsules were not merely a physical mixture of wall materials and core material. Furthermore, no new characteristic absorption peaks appeared in the microcapsules, suggesting that no new chemical bonds were formed during the complex coacervation process.

In addition, the amino absorption peak of gelatin at 3450 cm⁻¹ and the carboxyl absorption peak of gum Arabic at 1617 cm⁻¹ were both observed in the microcapsules, confirming effective interaction between the two wall materials. The FTIR results demonstrated that the characteristic absorption peaks of OEO were preserved in the microcapsules, and no new strong characteristic peaks were detected. These findings confirmed that the encapsulation of OEO within the gelatin–gum Arabic wall system occurred via physical entrapment without chemical reaction, while preserving the active structure of the essential oil.

### SEM observation

**Figure 3:**
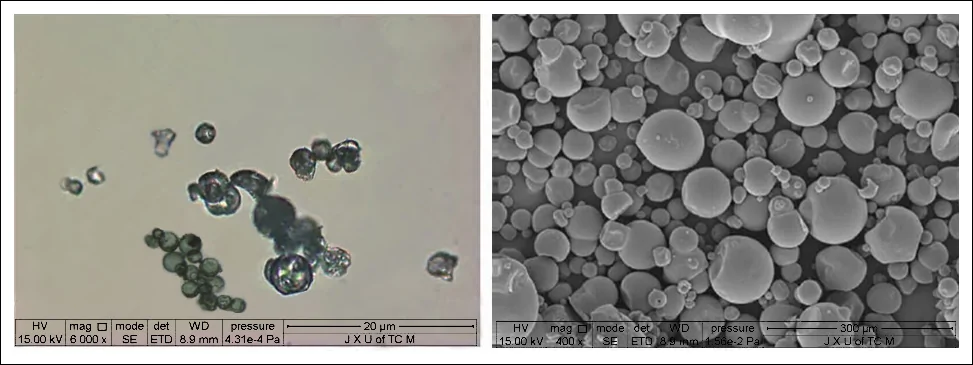
Scanning electron microscopic images of oregano essential oil microcapsules.

The surface morphology of freeze-dried microcapsules was observed by SEM, and the results are presented in Figure 3. The blank microcapsules (Panel A) exhibited surface depressions and lamellar aggregation, which may be attributed to shrinkage and collapse of the wall materials due to water evaporation from the microcapsule interior during freeze-drying, resulting in an irregular morphology. In contrast, OEO microcapsules (Panel B) displayed a relatively intact capsule wall structure with a smooth surface and no visible cavities or cracks.

This phenomenon may be explained by the ability of the transglutaminase to catalyze cross-linking among protein molecules through covalent bond formation with pectin molecules, thereby stabilizing the microstructure of the microcapsules. As shown in Panel B, the prepared OEO microcapsules were mononuclear, uniformly sized spherical particles with a particle diameter of approximately 110 nm.

## TGA

**Figure 4:**
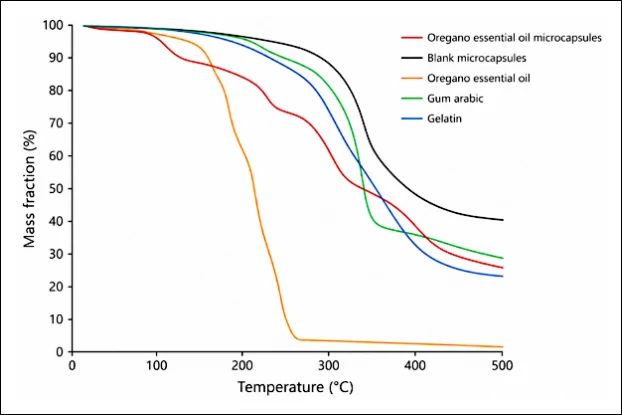
Thermogravimetric analysis curves of different microcapsules.

As shown in Figure 4, OEO began to decompose at approximately 125 °C, and its mass loss reached approximately 99% by 275 °C, indicating nearly complete volatilization. This result clearly reflected the highly volatile nature of the essential oil. In comparison, the wall materials exhibited much higher thermal stability. At approximately 250 °C, the mass losses of gelatin and gum Arabic were approximately 7% and 5%, respectively. At 500 °C, the residual mass fractions of gelatin and gum Arabic were 20% and 27%, respectively.

SEM observations further confirmed that OEO microcapsules prepared in this study exhibited spherical or subspherical structures with compact, smooth surfaces, without any obvious cracks or leakage. These findings indicated that the gelatin–gum Arabic complex coacervation system achieved complete encapsulation of OEO, favorable microcapsule formation, and stable microstructure.

Both OEO microcapsules and blank microcapsules exhibited a mass loss of approximately 1% within the range of 25 °C–100 °C. From 100 °C to 200 °C, the mass loss of blank microcapsules was approximately 3%, whereas that of OEO microcapsules was approximately 15%, compared with approximately 30% for pure OEO. Within 200 °C–350 °C, the mass losses of blank microcapsules and OEO microcapsules were approximately 45% and 42%, respectively. At 350–500 °C, both samples exhibited a mass loss of approximately 20%.

TGA demonstrated that the thermal stability of OEO microcapsules was markedly superior to that of free OEO. These results suggest that the gelatin–gum Arabic wall material effectively inhibited the volatilization and degradation of active ingredients, thereby providing favorable stability for application under high-temperature feed-processing conditions.

### DSC analysis

**Figure 5:**
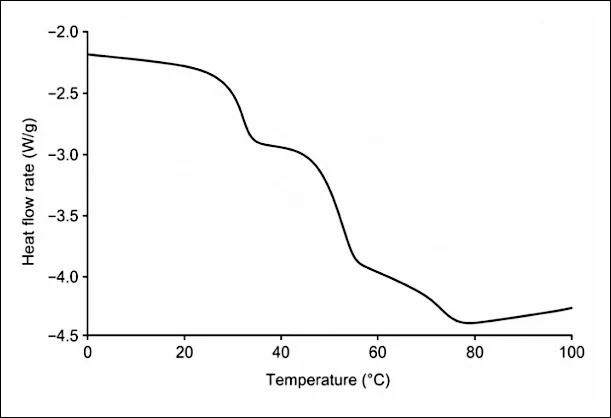
Differential scanning calorimetry curve of oregano essential oil microcapsules.

As presented in Figure 5, the glass transition temperature of OEO microcapsules was 50 °C, which was higher than normal room temperature (25 °C). DSC analysis showed that both the phase transition temperature and enthalpy of OEO were markedly improved after microencapsulation with gelatin–gum Arabic, further confirming that the microcapsule structure enhanced the thermal and structural stability of the essential oil.

### Sustained-release properties of OEO microcapsules

The release profiles of OEO from microcapsules were investigated at 5 °C, 26 °C, and 45 °C. After 70 days of sustained release, the retention rates of OEO in the microcapsules were 88.32%, 81.47%, and 78.53% at 5 °C, 26 °C, and 45 °C, respectively. These results indicated that higher temperature accelerated the release of OEO from microcapsules, resulting in lower retention rates.

### Effects of OEO microcapsules on growth performance of broilers

As shown in Table 2, compared with CK, no significant differences were observed in any measured indices during the starter phase (1–21 days of age) among all groups (p > 0.05). During the finisher phase (22–42 days of age), A1, A2, and A3 all promoted broiler growth relative to CK. Among these treatments, A3 showed the optimal effect, with final body weight and ADG significantly higher than those of CK (p < 0.05).

**Figure 6:**
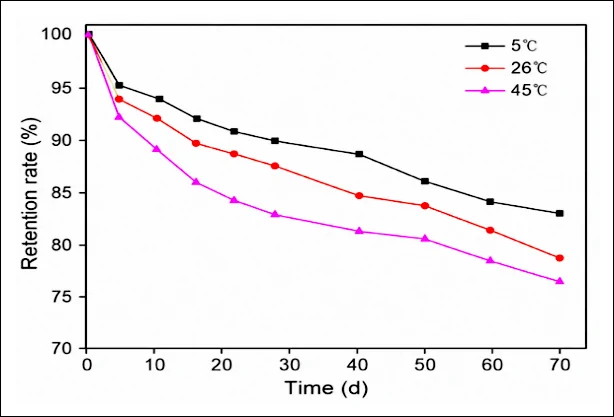
Effect of temperature on the retention rate of oregano essential oil microcapsules.

Over the entire experimental period (1–42 days of age), ADG increased with increasing dietary supplementation of OEO microcapsules. The ADG of A3 was significantly higher than that of CK (p < 0.05), whereas no significant difference was observed between A2 and A3 (p > 0.05) or between A1 and CK (p > 0.05). The F/G of A3 was significantly lower than that of CK (p < 0.05). In addition, mortality and culling rate decreased significantly with increasing inclusion levels of OEO microcapsules (p < 0.05).

**Table 2:** Effects of oregano essential oil microcapsules on growth performance of broilers.

**Age stage**	**Item**	**CK**	**A1**	**A2**	**A3**
1–21 days	Initial body weight (g)	41.37 ± 0.61	41.99 ± 0.66	40.88 ± 1.00	41.19 ± 0.28
	Final body weight (g)	909.73 ± 14.11	910.88 ± 25.21	922.10 ± 21.61	915.18 ± 22.21
	ADG, g	41.41 ± 0.67	43.44 ± 1.16	44.06 ± 1.14	43.69 ± 1.23
	ADFI, g	60.89 ± 2.52	60.96 ± 2.31	61.83 ± 1.30	60.76 ± 0.81
	F/G	1.40 ± 0.03	1.40 ± 0.02	1.40 ± 0.04	1.39 ± 0.04
22–42 days	Final body weight (g)	2463.27^b^ ± 56.90	2503.38^b^ ± 29.30	2527.49^ab^ ± 20.11	2577.72^a ^± 39.27
	ADG, g	77.67^b^ ± 2.15	79.62^ab ^± 1.27	80.26^ab^ ± 1.40	83.12^a^ ± 0.58
	ADFI, g	138.61 ± 1.65	139.45 ± 1.42	139.62 ± 1.13	140.83 ± 2.04
	F/G	1.78 ± 0.03	1.75 ± 0.06	1.74 ± 0.06	1.69 ± 0.07
1–42 days	ADG, g	56.02^b^ ± 1.33	58.97^b ^± 0.72	59.55^ab^ ± 0.43	60.73^a ^± 0.96
	ADFI, g	99.13 ± 1.82	99.45 ± 1.16	99.87 ± 1.26	100.74 ± 0.85
	F/G	1.77^a^ ± 0.04	1.69^ab ^± 0.05	1.68^ab^ ± 0.03	1.66^b^ ± 0.04
	Mortality and culling rate (%)	4.11^a^ ± 0.25	3.78^ab^ ± 0.42	3.11^bc^ ± 0.52	2.78^c^ ± 0.43

Note: Values with different lowercase superscript letters within the same row indicate significant differences (*p* < 0.05), whereas values with the same lowercase superscript letters or without superscript letters indicate no significant difference (*p* > 0.05). CK = Basal diet without supplementation, A1 = Basal diet supplemented with 80 mg/kg oregano essential oil microcapsules, A2 = Basal diet supplemented with 120 mg/kg oregano essential oil microcapsules, A3 = Basal diet supplemented with 160 mg/kg oregano essential oil microcapsules, ADG = Average daily gain, ADFI = Average daily feed intake, F/G = Feed-to-gain ratio. Means within the same row with different superscripts (a–c) differ significantly (*p* < 0.05).

Overall, OEO microcapsules prepared in this study improved growth performance, optimized slaughter performance, and enhanced meat quality and the nutritional composition of muscles in broilers. These findings indicate their promising potential for use as alternatives to antibiotics in green, healthy broiler production.

### Effects of OEO microcapsules on slaughter performance in broilers

As shown in Table 3, the breast muscle percentage of broilers in A3 was significantly higher than that in CK and A1 (p < 0.05). In addition, abdominal fat percentage in CK and A1 was significantly higher than that in A3 (p < 0.05). No significant differences were observed in dressing percentage, semi-eviscerated percentage, full-eviscerated percentage, or thigh muscle percentage among all treatment groups (p > 0.05).

**Table 3:** Effects of oregano essential oil microcapsules on slaughter performance of broilers.

**Item**	**CK**	**A1**	**A2**	**A3**
Dressing percentage (%)	91.04 ± 0.95	91.31 ± 0.25	91.78 ± 0.34	91.84 ± 0.57
Semi-eviscerated percentage (%)	84.58 ± 0.58	85.07 ± 0.10	85.20 ± 0.33	85.20 ± 0.35
Full-eviscerated percentage (%)	69.72 ± 0.61	70.36 ± 0.44	70.65 ± 0.61	70.66 ± 0.58
Breast muscle percentage (%)	24.12^b ^± 0.47	24.21^b^ ± 0.58	24.79^ab^ ± 0.27	25.20^a^ ± 0.25
Thigh muscle percentage (%)	17.71 ± 0.48	17.87 ± 0.31	17.98 ± 0.38	18.31 ± 0.38
Abdominal fat percentage (%)	2.37a ± 0.07	2.29a ± 0.06	2.25ab ± 0.04	2.16b ± 0.03

Values are presented as mean ± standard deviation. CK = Basal diet without supplementation, A1 = Basal diet supplemented with 80 mg/kg oregano essential oil microcapsules, A2 = Basal diet supplemented with 120 mg/kg oregano essential oil microcapsules, A3 = Basal diet supplemented with 160 mg/kg oregano essential oil microcapsules. Means within the same row with different superscripts (a,b) differ significantly (*p* < 0.05).

Dietary supplementation with OEO microcapsules significantly increased breast muscle percentage and decreased abdominal fat percentage in broilers, thereby improving slaughter performance. These results indicate that microencapsulating OEO with gelatin–gum Arabic may enhance its nutritional regulatory function and improve its economic value in livestock and poultry production.

### Effects of OEO microcapsules on meat quality of broilers

As shown in Table 4, *pH₁ₕ* and *pH₂₄ₕ *values of breast muscle in A3 were significantly higher than those in CK (p < 0.05). The a* value of breast muscle in A3 and drip loss of breast muscle in A2 and A3 were significantly lower than those in CK (p < 0.05). The L* value of thigh muscle in A3 was significantly lower than that in CK (p < 0.05).

**Table 4:** Effects of oregano essential oil microcapsules on meat quality of broilers.

**Item**	**CK**	**A1**	**A2**	**A3**
Breast muscle				
Lightness (L*)	59.60 ± 1.87	59.04 ± 1.23	58.29 ± 1.25	57.36 ± 1.31
Redness (a*)	3.11ᵇ ± 0.32	3.13ᵇ ± 0.17	3.38ᵃᵇ ± 0.15	3.70ᵃ ± 0.16
Yellowness (b*)	10.24 ± 0.48	9.95 ± 0.44	9.62 ± 0.62	9.47 ± 0.52
*p*H₁ₕ	6.18ᵇ ± 0.04	6.22ᵃᵇ ± 0.03	6.25ᵃᵇ ± 0.07	6.27ᵃ ± 0.02
*p*H₂₄ₕ	5.79ᵇ ± 0.03	5.83ᵃᵇ ± 0.01	5.83ᵃᵇ ± 0.02	5.85ᵃ ± 0.02
Shear force (*N*)	23.78 ± 1.28	23.51 ± 1.60	22.85 ± 1.92	21.80 ± 1.35
Drip loss (%)	2.46ᵃ ± 0.13	2.31ᵃᵇ ± 0.13	2.16ᵇ ± 0.09	2.11ᵇ ± 0.16
Thigh muscle				
Lightness (L*)	52.01ᵃ ± 0.77	51.30ᵃᵇ ± 0.86	49.91ᵃᵇ ± 1.46	49.76ᵇ ± 1.67
Redness (a*)	6.42ᵇ ± 0.27	6.86ᵃᵇ ± 0.54	7.11ᵃᵇ ± 0.45	7.56ᵃ ± 0.25
Yellowness (b*)	8.36 ± 0.85	8.21 ± 0.52	7.80 ± 0.60	7.49 ± 0.82
*p*H₁ₕ	6.26ᵇ ± 0.04	6.30ᵇ ± 0.04	6.33ᵃᵇ ± 0.03	6.38ᵃ ± 0.05
*p*H₂₄ₕ	5.83ᵇ ± 0.02	5.86ᵇ ± 0.01	5.90ᵃᵇ ± 0.05	5.93ᵃ ± 0.04
Shear force (*N*)	36.00 ± 2.42	35.94 ± 0.57	35.14 ± 2.63	34.81 ± 2.97
Drip loss (%)	1.44 ± 0.13	1.42 ± 0.14	1.39 ± 0.15	1.35 ± 0.13

Values are presented as mean ± standard deviation. CK = Basal diet without supplementation, A1 = Basal diet supplemented with 80 mg/kg oregano essential oil microcapsules, A2 = Basal diet supplemented with 120 mg/kg oregano essential oil microcapsules, A3 = Basal diet supplemented with 160 mg/kg oregano essential oil microcapsules, L* = Lightness, a* = Redness, b* = Yellowness,* p*H*₁ₕ* = Muscle *p*H measured at 1 h postmortem,* p*H₂₄ₕ = Muscle pH measured at 24 h postmortem. Means within the same row with different superscripts (a,b) differ significantly (*p* < 0.05).

Dietary supplementation with OEO microcapsules improved meat color, enhanced water-holding capacity, reduced drip loss, and increased tenderness of broiler meat, thereby improving overall meat quality. These results suggest that supplementation positively affects the commercial value of broiler meat, possibly through antioxidant activity and regulation of nutrient metabolism.

### Effects of OEO microcapsules on proximate nutritional composition of broiler meat

As shown in Table 5, crude protein content in breast muscle was significantly higher in A3 than in CK (p < 0.05), whereas crude fat content was significantly lower (p < 0.05). Crude fat content in thigh muscle decreased significantly with increasing dietary supplementation of OEO microcapsules (p < 0.05).

**Table 5:** Effects of oregano essential oil microcapsules on proximate nutrient composition of broiler meat.

**Item**	**CK**	**A1**	**A2**	**A3**
**Breast muscle**				
Crude protein (%)	22.79ᵇ ± 0.50	23.32ᵃᵇ ± 0.46	23.73ᵃ ± 0.56	24.21ᵃ ± 0.43
Crude fat (%)	1.57ᵃ ± 0.07	1.47ᵃᵇ ± 0.05	1.44ᵇ ± 0.08	1.39ᵇ ± 0.08
Moisture (%)	74.57 ± 0.98	74.20 ± 1.17	74.11 ± 0.76	74.04 ± 0.90
Ash (%)	1.16 ± 0.07	1.18 ± 0.09	1.16 ± 0.06	1.17 ± 0.05
**Thigh muscle**				
Crude protein (%)	20.24 ± 0.32	20.19 ± 0.57	20.56 ± 0.54	20.87 ± 0.50
Crude fat (%)	4.54ᵃ ± 0.32	4.14ᵃᵇ ± 0.29	3.78ᵇ ± 0.38	3.57ᵇ ± 0.45
Moisture (%)	73.10 ± 0.45	73.06 ± 0.41	72.95 ± 0.25	72.76 ± 0.37
Ash (%)	1.28 ± 0.04	1.26 ± 0.07	1.28 ± 0.07	1.29 ± 0.05

Values are presented as mean ± standard deviation. CK = Basal diet without supplementation, A1 = Basal diet supplemented with 80 mg/kg oregano essential oil microcapsules, A2 = Basal diet supplemented with 120 mg/kg oregano essential oil microcapsules, A3 = Basal diet supplemented with 160 mg/kg oregano essential oil microcapsules. Means within the same row with different superscripts (a,b) differ significantly (p < 0.05). Crude protein, crude fat, moisture, and ash contents are expressed as percentages (%).

Dietary supplementation with OEO microcapsules significantly increased crude protein content, reduced crude fat deposition in broiler breast muscle, and optimized the proximate nutritional composition of muscle. These findings indicate that OEO microcapsules can improve the nutritional value of chicken meat and exert favorable nutritional regulatory effects.

## DISCUSION

### Preparation and characterization of OEO microcapsules

In this study, OEO microcapsules were successfully prepared through complex coacervation using gelatin and gum Arabic as wall materials, followed by comprehensive multidimensional characterization. Gelatin and gum Arabic, which carry opposite charges, formed a stable microcapsule wall system with good biocompatibility and biodegradability, enabling efficient encapsulation and protection of OEO. Morphological observations showed that the microcapsules had relatively regular spherical structures with compact and intact surfaces, without obvious cracks or leakage, indicating well-controlled complex coacervation and effective encapsulation of the core material by the wall matrix.

FTIR analysis confirmed that the characteristic absorption peaks of OEO were preserved in the microcapsules without the appearance of new strong characteristic peaks. This finding suggests that the interaction between the wall and core materials was primarily physical entrapment rather than an irreversible chemical reaction, thereby preserving the integrity of the essential oil's active components. TGA demonstrated that microencapsulation significantly improved the thermal stability of OEO and effectively inhibited the loss of volatile components, providing reliable stability for feed processing, high-temperature pelleting, and related industrial applications.

Overall, the characterization results verified that gelatin–gum Arabic complex coacervation microcapsules improved the stability, dispersibility, and controlled-release properties of OEO. This provides a feasible technical strategy to overcome limitations such as high losses and inconsistent efficacy of natural essential oils as antibiotic alternatives in livestock and poultry production and lays a material foundation for the development of green, safe, and efficient antibiotic-free feed additives [16].

### Effects of OEO microcapsules on growth performance of broilers

Dietary supplementation with OEO microcapsules improved ADG, reduced F/G, and enhanced overall growth performance in broilers. This growth-promoting effect can be explained by four major mechanisms: sustained-release activity, antibacterial and anti-inflammatory effects, improved intestinal health, and enhanced digestion and absorption. In the present study, microencapsulated OEO had no significant effect on growth performance during the starter phase, whereas supplementation at 160 mg/kg significantly increased final body weight and overall ADG and reduced F/G, mortality, and culling rate.

The underlying mechanism may relate to microencapsulation’s ability to overcome the limitations of free OEO, including high volatility, susceptibility to oxidation, and poor stability. Microencapsulation enables targeted, slow, and sustained release of active ingredients, primarily carvacrol and thymol, in the gastrointestinal tract, thereby maximizing their bioavailability. These active components can inhibit colonization and proliferation of intestinal pathogenic bacteria, including *Escherichia coli *and *Salmonella* spp*.*, promote beneficial bacteria such as lactic acid bacteria, and optimize intestinal microbial balance. They may also increase intestinal villus height, reduce crypt depth, enhance the villus height-to-crypt depth ratio, strengthen the intestinal absorptive area and barrier function, and reduce nutrient loss due to intestinal inflammation and oxidative stress.

In addition, OEO may stimulate digestive enzyme secretion, improve apparent nutrient digestibility, enhance feed palatability through its aromatic odor, and moderately increase feed intake, thereby accelerating weight gain and reducing F/G. The beneficial effects of OEO microcapsules on growth performance and mortality reduction observed in this study can therefore be attributed to improved intestinal health and microbial balance.

Previous research reported that feed conditioning temperature and time significantly affect pellet quality, ileal morphology, microbial flora, and metabolizable energy, and reviews have indicated that feed conditioning parameters influence broiler growth performance by regulating feed quality and intestinal health [17, 18]. In agreement with these observations, the present study further supports that OEO microcapsules improve nutrient absorption efficiency and systemic health by optimizing intestinal morphology, improving ileal microbial balance, and enhancing intestinal barrier integrity, ultimately improving growth performance and reducing mortality.

A significant reduction in broiler mortality was observed in the high-dose OEO microcapsule group, suggesting favorable effects on body protection and health improvement. Although immune, oxidative stress, and toxin-response biomarkers were not measured in the present experiment, the observed biological responses can be reasonably explained by improved intestinal structural integrity, enhanced nutrient absorption, and reduced intestinal damage. Collectively, these findings support the role of OEO microcapsules in enhancing immunity, alleviating oxidative and potential intestinal injury, and reducing mortality. The results are consistent with previous domestic and international reports indicating that OEO microcapsules can replace antibiotic growth promoters and improve broiler growth performance. Therefore, OEO microcapsules prepared with gelatin–gum Arabic wall materials may exert antibiotic-alternative and growth-promoting effects through stable encapsulation, targeted release, and multitarget regulation, providing a safe, efficient, and stable green additive strategy for antibiotic-free broiler production.

### Effects of OEO microcapsules on slaughter performance of broilers

Slaughter performance is a key indicator for evaluating economic value and nutrient deposition efficiency in broilers. In this study, dietary supplementation with 160 mg/kg OEO microcapsules significantly increased breast muscle percentage and decreased abdominal fat percentage. These findings are consistent with previous studies. However, the dressing percentage, semi-eviscerated percentage, and full-eviscerated percentage did not improve significantly, which may be attributed to differences in breed, feeding environment, slaughtering techniques, or sampling variation.

Mechanistically, the positive effects of OEO microcapsules on slaughter performance may be attributed to three major pathways. First, OEO microcapsules improve intestinal health and nutrient partitioning by inhibiting harmful intestinal bacteria, maintaining microbial stability, and improving intestinal villus morphology and barrier function. These effects enhance feed conversion efficiency and reduce nutrient losses due to intestinal inflammation and oxidative stress, allowing more nutrients to be deposited in muscle rather than fat.

Second, carvacrol and thymol may regulate metabolism and immune responses by scavenging free radicals and alleviating metabolic stress by activating antioxidant enzyme systems. These compounds may also improve immune organ indices and enhance nonspecific immunity, thereby reducing disease-related nutrient consumption and promoting lean meat deposition.

Third, the controlled-release and synergistic effects of microcapsules may enhance the stability and persistence of biological activity. Compared with free essential oil, the gelatin–gum Arabic wall material provides sustained release and protects active ingredients through complex coacervation, reduces gastrointestinal irritation caused by high concentrations of essential oil, and ensures stable absorption and long-term activity in the digestive tract. These processes may explain the sustained growth-promoting and muscle-depositing effects observed in the present study.

### Effects of OEO microcapsules on meat quality of broilers

OEO microcapsules exerted beneficial effects on the sensory and physicochemical qualities of broiler meat. Dietary supplementation improved meat color by increasing redness (a*) and enhanced water-holding capacity by reducing drip loss and shear force while maintaining appropriate *p*H values.

The improvement in meat quality may be attributed to three mechanisms. First, the antioxidant activity of encapsulated carvacrol and thymol may reduce free radical accumulation, inhibit lipid and protein oxidation in muscle tissue, delay muscle fiber denaturation and meat discoloration, and maintain color stability. Second, improved intestinal health may enhance nutrient distribution by optimizing microbial composition and villus morphology, reducing nutrient loss from intestinal inflammation, promoting muscle protein deposition, decreasing abdominal fat percentage, and increasing lean meat percentage and meat compactness. Third, controlled release from the gelatin–gum Arabic wall material may reduce gastrointestinal irritation caused by concentrated essential oils, support stable absorption of active ingredients, and provide sustained antioxidant and antibacterial effects.

This study showed that OEO microcapsules significantly reduced abdominal fat deposition and increased breast muscle percentage in broilers, while increasing crude protein content and decreasing crude fat content in breast muscle. These findings indicate that OEO may regulate lipid metabolism and nutrient partitioning. Previous poultry nutrition studies have confirmed that plant-derived active substances can regulate lipid metabolism, cholesterol levels, and muscle composition by modulating metabolic pathways, thereby improving fat deposition and protein deposition efficiency. These observations provide theoretical support for the role of OEO microcapsules in regulating lipid catabolism, reducing abdominal fat accumulation, and promoting protein synthesis and muscle deposition.

### Effects of OEO microcapsules on proximate nutritional composition of broiler meat

Dietary supplementation with OEO microcapsules significantly altered the proximate nutritional composition of broiler muscle. Supplementation with 160 mg/kg OEO microcapsules significantly increased crude protein content in breast muscle while reducing crude fat content. In addition, crude fat content in thigh muscle decreased significantly with increasing OEO supplementation, collectively improving the nutritional value and eating quality of broiler meat.

After encapsulation, OEO showed enhanced stability, and its active components, carvacrol and thymol, were steadily released in the intestinal tract. By regulating intestinal microbial balance, improving intestinal morphology, and enhancing nutrient digestibility and absorption, these compounds may promote amino acid uptake and protein anabolism, thereby increasing crude protein deposition in muscle. In addition, OEO may modulate lipid metabolism, suppress excessive fat deposition, and reduce crude fat content in muscle, thereby making the nutritional profile of broiler meat more consistent with consumer demand for healthier meat products.

The protective and sustained-release properties of the microcapsule wall material helped overcome the limitations of free OEO, including high volatility, strong irritancy, and low bioavailability, resulting in more stable and durable regulatory effects on muscle nutritional composition. These findings are consistent with previous studies showing that encapsulated OEO increases muscle protein content in broilers by improving metabolism and intestinal health. Additional studies have confirmed that sustained-release plant essential oils optimize nutritional composition, enhance the nutritional value of broiler meat, and improve nutrient deposition in muscle.

Collectively, OEO microcapsules optimized the nutritional profile of broiler meat by enhancing nutrient utilization and regulating protein and lipid metabolism, suggesting their potential as a green additive to improve meat quality. In summary, OEO exerts beneficial effects on intestinal function and overall poultry performance by enhancing growth performance, improving intestinal histomorphology, maintaining intestinal barrier integrity, and strengthening immunity and stress resistance. This study evaluated broilers from 22 to 42 days of age, suggesting that continuous supplementation with OEO microcapsules at 160 mg/kg until market age may be recommended [19, 20].

### Strengths, limitations, and future perspectives

Microencapsulated OEO was prepared using complex coacervation with gelatin and gum Arabic as wall materials. This study systematically evaluated the effects of OEO microcapsules on growth performance, carcass traits, meat quality, and nutritional composition in broilers. The preparation method offers advantages, including high encapsulation efficiency, a stable structure, protection of active components, and suitability for industrial applications in livestock feed. These findings provide a reliable technical reference for developing plant essential oil-based alternatives to antibiotic growth promoters.

However, this study has several limitations. First, it evaluated the effects over a single production cycle and did not investigate responses under different environmental stresses or rearing models. Second, immune, oxidative stress, microbiota, and intestinal morphology biomarkers were not measured, limiting direct mechanistic interpretation. Third, the molecular mechanisms and metabolic pathways underlying the regulatory effects of OEO microcapsules on broiler growth, intestinal health, meat quality, and nutrient metabolism remain unclear.

Future studies should conduct multibatch, long-term field trials under different production systems and environmental conditions. In addition, integrated omics approaches, intestinal microbiome profiling, evaluation of antioxidant and immune biomarkers, and histomorphological assessment should be used to identify target sites and molecular mechanisms of action. Such studies would provide more comprehensive scientific evidence for the large-scale application of OEO microcapsules in antibiotic-free livestock farming [21–23].

## CONCLUSION

This study successfully developed OEO microcapsules using gelatin–gum Arabic complex coacervation and demonstrated that the microencapsulation process provided high encapsulation efficiency, improved thermal stability, and favorable sustained-release characteristics. Dietary supplementation with microencapsulated OEO, particularly at 160 mg/kg, significantly enhanced growth performance by increasing final body weight and ADG, reducing F/G, and lowering mortality and culling rates in broilers. In addition, supplementation improved carcass characteristics by increasing breast muscle percentage and decreasing abdominal fat deposition. Meat quality was also enhanced through improvements in pH, water-holding capacity, and color characteristics, accompanied by increased crude protein content and reduced crude fat content in breast and thigh muscles, thereby improving the nutritional value of broiler meat.

From a practical perspective, these findings indicate that microencapsulated OEO represents a promising phytogenic feed additive and a viable alternative to antibiotic growth promoters in antibiotic-free broiler production. The controlled-release properties and enhanced stability achieved through microencapsulation offer advantages for feed processing and commercial applications, contributing to sustainable, environmentally friendly poultry production.

In conclusion, microencapsulation effectively enhanced the stability and functionality of OEO, and dietary supplementation with microencapsulated OEO improved growth performance, carcass characteristics, meat quality, and nutrient deposition in broilers. These findings support the potential of microencapsulated OEO as a safe, efficient, and sustainable green feed additive for modern poultry production systems.

## DATA AVAILABILITY

The data generated during the study are included in the manuscript.

## GENERATIVE AI DECLARATION

The authors declare that generative artificial intelligence (AI) tools were used solely to improve language, grammar, and readability during manuscript preparation. All scientific content, data analysis, interpretation of results, and conclusions were developed and verified by the authors. The authors take full responsibility for the accuracy, integrity, and originality of the work presented, and no AI tool was listed as an author.

## AUTHORS’ CONTRIBUTIONS

CL and JE: Conceived, designed, and coordinated the study and developed the data collection tools. LY and TL: Supervised field sampling, data collection, laboratory analyses, and data entry. LJ and XZ: Managed the project, performed statistical analyses and data interpretation, prepared the original manuscript draft, and developed the visualizations. LY and XZ: Coordinated the study, critically reviewed and revised the manuscript, and ensured compliance with ethical standards. All authors have read and approved the final version of the manuscript.
